# Supplementary figures and images for: Cellular determinants of parvovirus B19 susceptibility in the human placenta
Source: PLoS Pathog. 2026 Feb 18;22(2):e1013984. doi: 10.1371/journal.ppat.1013984 (PMC12928586; doi:10.1371/journal.ppat.1013984)

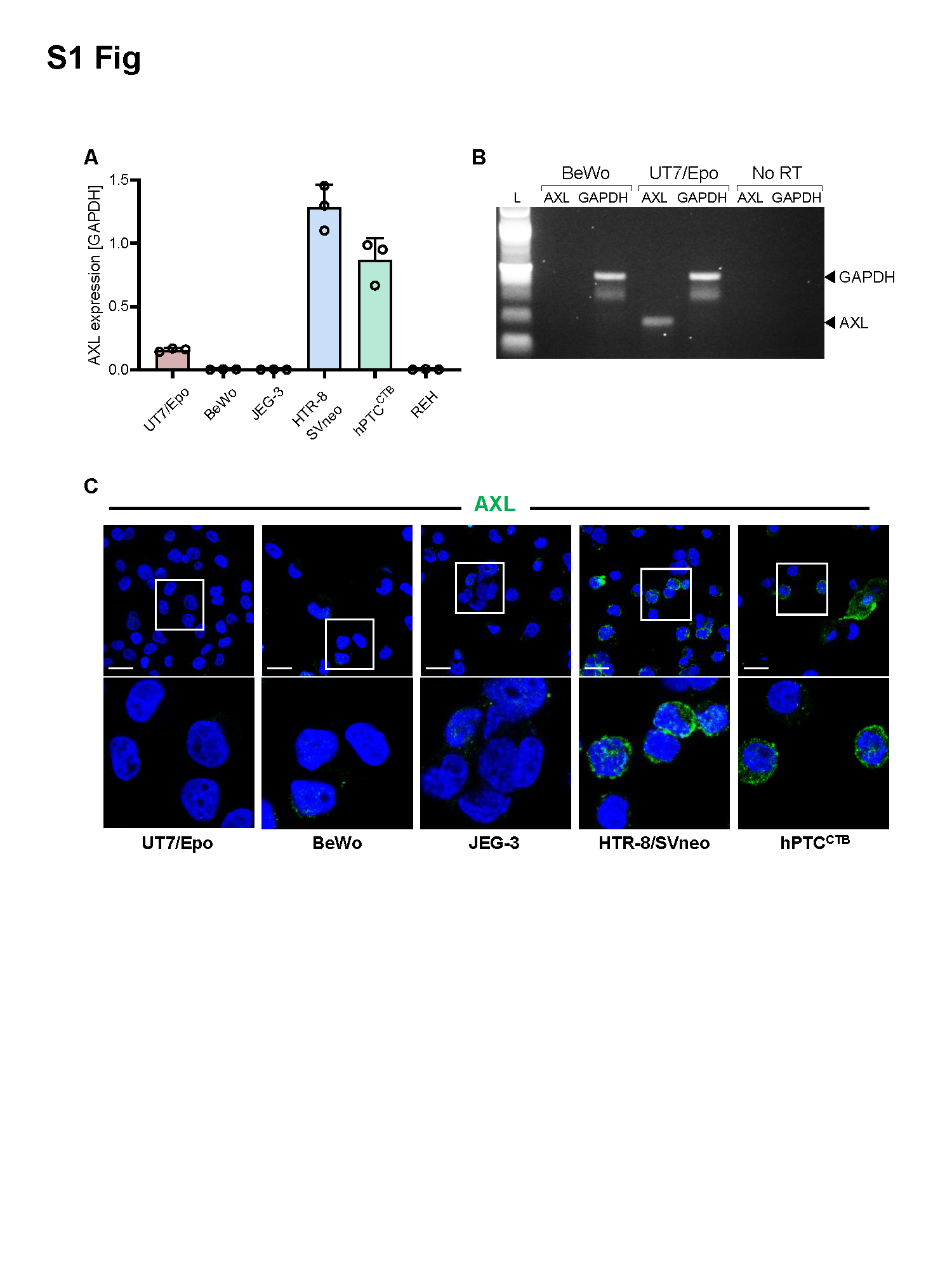

Supplement: S1 Fig — (A) Relative expression of AXL mRNA in UT7/Epo and in different trophoblast subtypes. mRNA levels were measured by RT-qPCR and normalized to GAPDH mRNA. (B) RT-PCR amplicons from BeWo and UT7/Epo cells were visualized by agarose gel electrophoresis. GAPDH, loading control; No RT, no reverse transcriptase; L, 1 kb DNA ladder. (C) Immunostaining of UT7/Epo and trophoblasts with an anti-AXL antibody (green). DAPI (Blue). Scale bar, 20 μm. All results are presented as the mean ± SD of three independent experiments. (TIF) [file ppat.1013984.s001.tif]

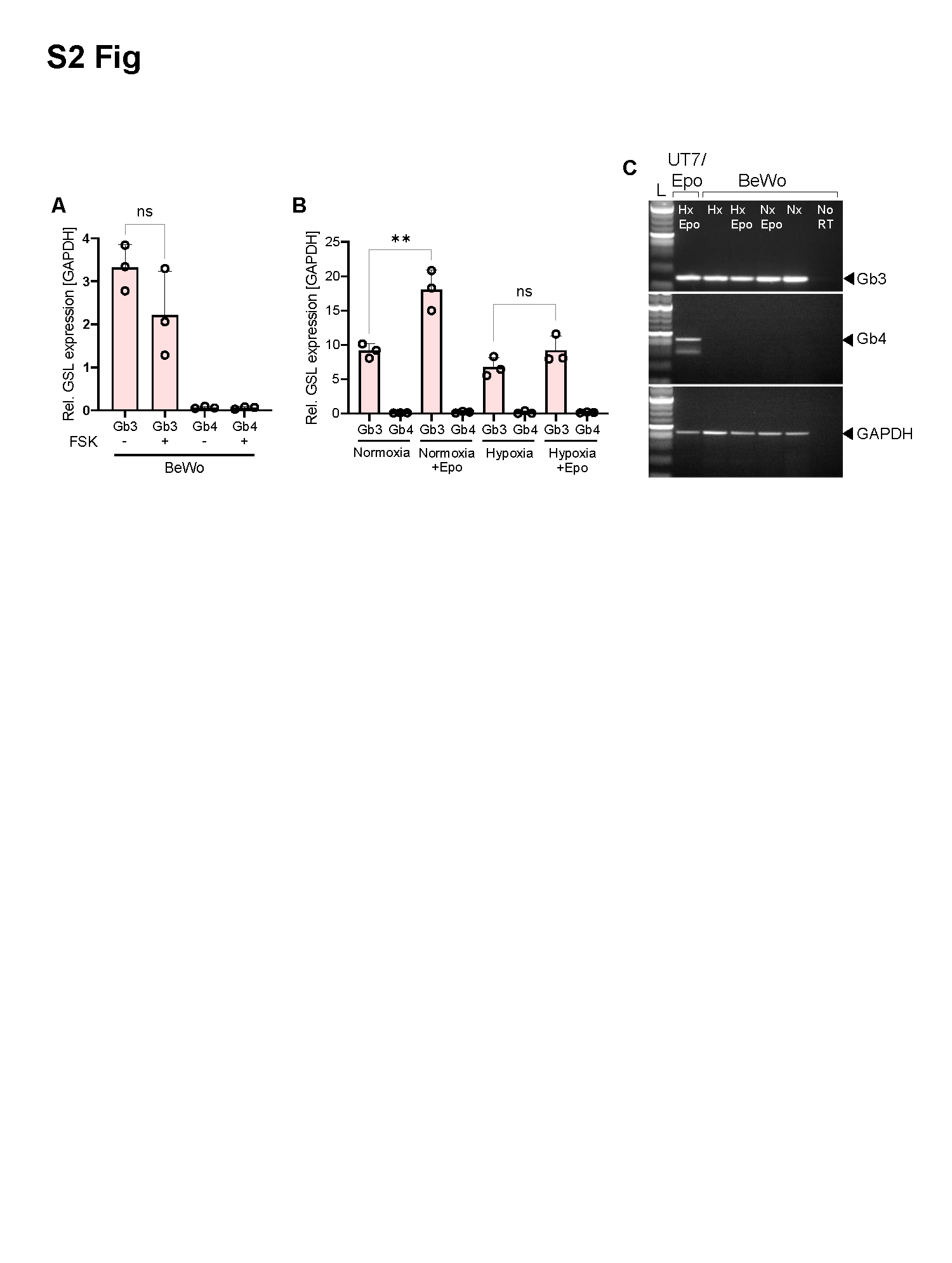

Supplement: S2 Fig — (A) Relative mRNA expression of Gb3 and Gb4 in BeWo cells treated or untreated with forskolin (FSK), normalized to GAPDH mRNA, to assess the effect of syncytiotrophoblast differentiation on Gb3/Gb4 expression. (B) Relative mRNA expression of Gb3 and Gb4 in BeWo cells exposed to normoxia, normoxia plus Epo, hypoxia, or hypoxia plus Epo, normalized to GAPDH mRNA, to assess the effect of oxygen concentration and Epo on Gb3/Gb4 expression. (C) Agarose gel electrophoresis of RT-PCR products corresponding to Gb3, Gb4, and GAPDH in BeWo cells under the conditions described in (B), and in UT7/Epo cells incubated with or without Epo under normoxia. Each panel displays the PCR products for Gb3, Gb4, or GAPDH as indicated. No RT, no reverse transcriptase; 1 kb DNA ladder. All results are presented as the mean ± SD of three independent experiments. Statistical significance was calculated using two-sided Student’s t-test. **p < 0.05; ns, non-significant. (TIF) [file ppat.1013984.s002.tif]

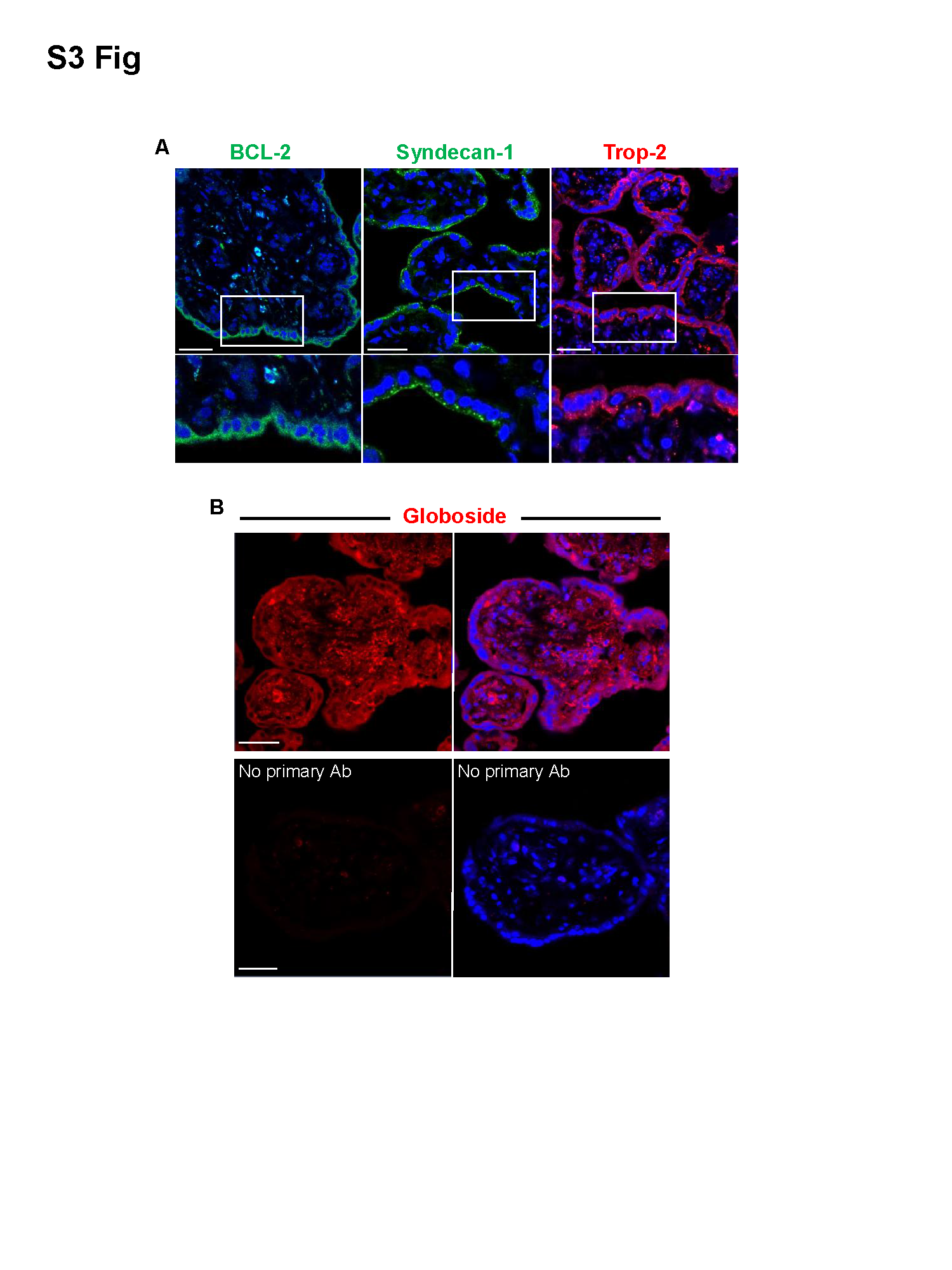

Supplement: S3 Fig — (A) Trophoblast markers BCL-2, Trop-2 (both in green), and CD138 (red), detected in term placenta cryosections. DAPI (Blue). Scale bar, 40 μm. (B) Globoside (red), detected in term placenta cryosections. DAPI (Blue). Scale bar, 40 μm. (TIF) [file ppat.1013984.s003.tif]

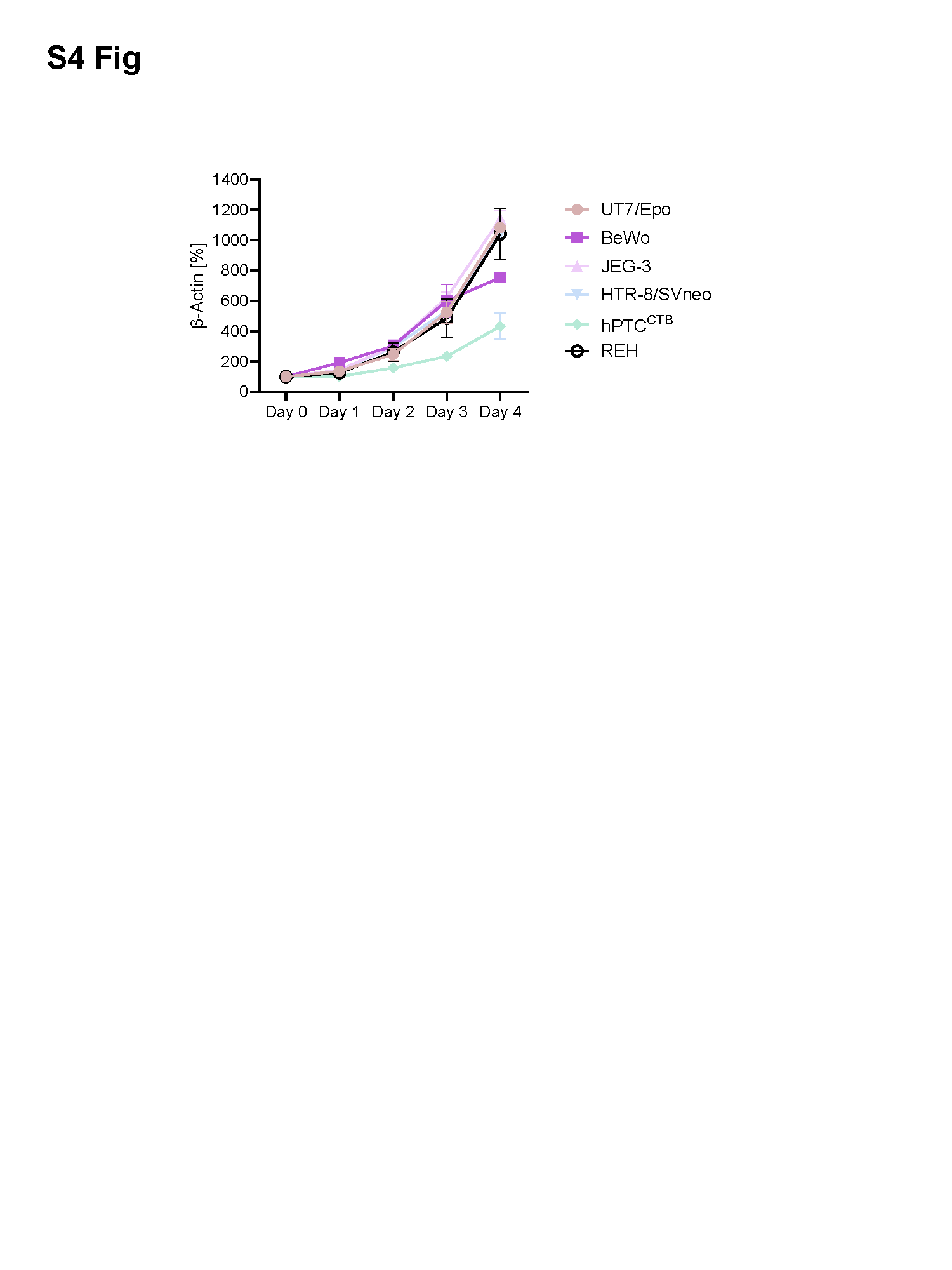

Supplement: S4 Fig — Relative β-actin DNA levels were quantified by RT-qPCR in different trophoblast subtypes, UT7/Epo and REH cells, at days 1, 2, 3, and 4 of culture. Data are expressed as a percentage of β-actin expression relative to day 0. All results are presented as the mean ± SD of three independent experiments. (TIF) [file ppat.1013984.s004.tif]
