# Supplementary material for: Cellular determinants of parvovirus B19 susceptibility in the human placenta
Source: PLoS Pathog. 2026 Feb 18;22(2):e1013984. doi: 10.1371/journal.ppat.1013984 (PMC12928586; doi:10.1371/journal.ppat.1013984)
Supplement: Attachment — Submitted filename: Response to Reviewers.docx [file ppat.1013984.s007.docx]

**Response to Reviewers**

**Part I - Summary**
Please use this section to discuss strengths/weaknesses of study, novelty/significance, general execution and scholarship.

Reviewer #1: In the manuscript entitled " Cellular determinants of parvovirus B19 infection in the human placenta", Suter and collogues employ a wide range of sophisticated techniques developed by the Ros laboratory over the years, to address the issue of B19V ability to infect different placenta cell types.
The topic is of great interest, given the ability of B19V to cause severe disease and even death of the fetus in cases of congenital infection. Given the absence of approved, safe, and efficient antivirals, B19V congenital infection represents a major medical problem. Since B19V is believed to productively infect exclusively EPCs, it is not clear how fetal infection would occur, and the ability of the virus to infect the placenta is still unknown.

To address this issue the authors first assess the ability of VP1 (the viral attachment protein) to bind to the cell surface of several cell types of placental origin. For certain cell types the authors clearly show efficient binding, that suggests the ability to interact with cell surface constituents. Secondly, the ability of VP1 expressing VLPs to be internalized is shown, further supports the possibility that B19V might interact with placental cell types. Subsequently, the authors show that after infection or transfection certain cell types allow expression of structural protein mRNA.

Based on such evidences, the authors conclude that specific placental cell types express the still-unidentified Vp1 receptor (VP1uR) and are permissive to B19V replication.

While the study addresses an interesting question and data is of high quality, its interpretation suffers from two major ambiguities that, in my view, significantly affect the strength and balance of the conclusions.

First, the authors frequently associate the ability of certain cell types to bind VP1-containing particles with the presence of a putative VP1 receptor (VP1uR). Given that VP1uR remains unidentified at the molecular level, this interpretation is speculative and should be presented with greater caution. The observed binding or uptake could reflect non-specific interactions or alternative entry mechanisms. I do not think it is possible to prove the expression of an unidentified factor.

We agree that, more than a specific receptor, VP1uR is better defined as a factor or molecular entity that supports VP1u binding and uptake. VP1uR is defined functionally by its strict, RBD-dependent interaction with VP1u and its precise correlation with B19V uptake.

In our assays, only VP1u constructs with an intact RBD (recVP1u and MS2-VP1u) bind and internalize in trophoblasts. The receptor-binding deficient mutant ΔN29, shows no binding or uptake in any cell type, ruling out nonspecific interactions. This pattern mirrors native B19V binding and uptake in all models tested. EVTs provide a clean negative control, lacking VP1u-mediated uptake and, consequently, B19V uptake is not detected.

These results align with extensive prior work, where VP1u-dependent internalization and ΔN29 controls have been repeatedly validated as a highly specific readout of VP1uR activity.

Thus, while the receptor molecule remains unidentified, our data provides functional evidence for a receptor supporting B19V VP1u interaction and uptake, which we refer to as VP1uR for simplicity.

**Actions**: To avoid confusion, we have defined VP1uR the first time that is mentioned in the Introduction (lines 79-81) as “*molecular entity or complex targeted by the VP1u RBD that mediates viral binding and uptake*”. Throughout the manuscript and whenever possible, we replaced “receptor” wording with binding/uptake-based terminology to better reflect an activity and not the expression of a specific receptor.

Second, the authors equate detection of VP1 mRNA with cellular permissiveness to B19V infection. Expression of viral transcripts alone does not demonstrate productive infection, which can only be established by showing viral DNA replication and production of infectious progeny.

We agree with the reviewer that viral transcript detection does not by itself establish productive infection, which implies the generation of progeny infectious particles. Our data do not claim permissiveness in the trophoblasts models presented. Instead, we demonstrate susceptibility (virus entry) of specific trophoblast subtypes and identify cellular determinants that are critical for the infection to progress.

As shown in Fig. 5, BeWo and JEG-3 cells, although able to bind and internalize VP1u and to support virus uptake, are non-permissive because they lack globoside, an essential factor for B19V endosomal escape. Accordingly, NS1 and VP1/VP2 transcripts remain close to the background signal or undetectable, respectively. HTR-8/SVneo (EVTs) are likewise non-permissive because they lack VP1uR and, consequently, cannot internalize the virus. hPTC^CTB^ cells (term placenta) express both VP1uR and globoside, permitting viral entry, but exhibit only low NS1 transcription and barely detectable VP1/VP2 mRNAs due to their intrinsically low proliferative state. This is consistent with the requirement for active cell proliferation to support parvovirus DNA replication and structural gene expression.

**Actions:** We have revised the manuscript to clearly distinguish susceptibility (viral entry), transcriptional activity and permissiveness (full replication cycle).

Reviewer #2: This paper is a very interesting and important piece of work and a nice follow-up of their excellent previous work on the parvovirus B19 pH-dependent receptor switch and cell entry. This study shows an immense workload with many angles and different kinds of experiments to prove that B19V targets placental trophoblasts and uses a VP1u-specific (yet unknown) receptor, VP1uR, to enter placenta-derived cell lines and another receptor, globoside, to be released from the endosomes, leading to nuclear expression of its genes. They also show which placental-derived cells have either or both of these two receptors and which do not. This paper brings novel insights on how B19V infects the placenta in pregnancy. However, there are a few issues that will need clarification, outlined in Parts II and III below.

Reviewer #3: In this manuscript by Suter et al. entitled “Cellular determinants of parvovirus B19 infection in the human placenta”, the authors analyze in detail the susceptibility of three trophoblast cell types to infection by human parvovirus B19. The viral cellular entry and infection in cells were analyzed using detection of B19V receptor, binding and internalization assays, combined with confocal microscopy. The results show that trophoblast cells from the maternal-fetal interface, located between the fetal placenta and the maternal uterus, enable B19V infection. These findings expand the tropism of B19V to include trophoblast cells, thereby increasing our understanding of how the virus can cross the placenta and infect the fetus. The data are novel and informative, and the conclusion will be of interest to the virology, medical, and cell biology communities, as well as a broad audience.

**Part II – Major Issues: Key Experiments Required for Acceptance**
Please use this section to detail the key new experiments or modifications of existing experiments that should be absolutely required to validate study conclusions.

Generally, there should be no more than 3 such required experiments or major modifications for a "Major Revision" recommendation. If more than 3 experiments are necessary to validate the study conclusions, then you are encouraged to recommend "Reject".

Reviewer #1: The interpretative issues mentioned above introduce an element of overstatement in the manuscript’s narrative. While the experimental observations are interesting, (I) the conclusions regarding receptor usage should be substantially tempered to ensure they are consistent with the data presented. Secondly (II), permissiveness of placental cell types should be experimentally demonstrated by showing an increase over time of viral DNA, and by the production of viral progeny. Finally (III) attempts to characterize viral entry in placental cell types should be performed.

Specific comments
• Figure 5. The data shown is interesting, however the authors evaluated B19V gene expression exclusively at the mRNA level and only at 2, 24 and 48 h post-infection. I strongly recommend performing immunofluorescence (IF) or Western blotting analyses to confirm protein expression of key viral markers (e.g., VP1/VP2, NS1) expanding the time line to 120 h. In parallel, quantification of viral genomic DNA at both 48 h and 120 h post-infection is essential to assess viral replication. Demonstration of viral progeny production should be attempted. Further the entry mechanism has not been characterized here. The authors are world leaders in B19V entry and should attempt at least some initial characterization to compare the entry pathway between placental cell types and UT7E cells, as shown in (PMID: 22718826).

We appreciate the reviewer’s detailed comments and the opportunity to clarify these points. The non-permissive nature of the trophoblast models analyzed, as well as the specific stage(s) at which B19V infection is restricted in each cell type, are documented in the original manuscript through receptor analyses, viral entry assays, and time-resolved viral gene expression data. However, we acknowledge that these conclusions may not have been sufficiently emphasized and have revised the text to make them more explicit.

To clarify:

• BeWo and JEG-3 (first-trimester choriocarcinoma CTBs)

- VP1uR: present - Globoside: absent - Proliferation: high
- Consequence: virus internalizes but cannot escape endosomes.
- mRNA profile: NS1 undetectable or extremely low; VP1/VP2 not detected.

• HTR-8/SVneo (EVTs)

- VP1uR: absent - Globoside: present - Proliferation: high
- Consequence: virus cannot enter.
- mRNA profile: no NS1; no VP1/VP2.

• hPTC^CTB^ (term CTBs)

- VP1uR: present - Globoside: present - Proliferation: very low
- Consequence: virus enters but cannot undergo DNA replication.
- mRNA profile: modest NS1; barely detectable VP1/VP2, which is consistent with the requirement for robust DNA replication to access the VP1/VP2 coding region.

Importantly, the combined results clearly show that B19V infection requires the simultaneous presence of all three determinants. Among the placenta trophoblasts, only first-trimester villous trophoblasts naturally fulfill this triad. This convergence strongly supports the conclusion that early villous CTBs and their syncytial derivatives represent the susceptible compartment of the placental barrier.

**Actions:** Stated at the end of this result section (lines 287-289), “*Together, these results demonstrate that none of the trophoblast cell types analyzed are permissive to productive B19V infection, as each lack at least one essential determinant, i.e., VP1uR, globoside, or sufficient proliferative activity*”. Accordingly, the requested assays intended to demonstrate permissiveness beyond virus entry and transcription, such as viral protein expression, DNA replication, and infectious progeny production are not experimentally feasible in these cells.

• Figure 6. The data presented are not fully convincing. The authors evaluate B19V gene expression exclusively at the mRNA level and only at 48 h post-infection. While these results indicate transcriptional activity, they do not demonstrate productive viral replication. I strongly recommend performing immunofluorescence (IF) or Western blotting analyses to confirm protein expression of key viral markers (e.g., VP1/VP2, NS1).

In addition, quantification of viral genomic DNA at both 48 h and 120 h post-infection is essential to assess viral replication dynamics. A productive infection should be reflected by a clear increase in viral DNA copy number over time.

The purpose of Figure 6 was not to demonstrate productive parvovirus B19 (B19V) infection, but to assess whether the viral P6 promoter is transcriptionally active in trophoblast cells. We have clarified in the revised manuscript that the transfection strategy was specifically designed to test P6 promoter activity, rather than to model productive viral replication. To better mimic the natural infection, cells were transfected with B19V ssDNA genomes (not with a plasmid-derived dsDNA infectious clone). Because NS1 is transcribed exclusively from the P6 promoter, accumulation of NS1 mRNA directly reflects P6 promoter activity and demonstrates its functionality in trophoblast cells.

We additionally quantified VP1/VP2 mRNA. In B19V, efficient and biologically meaningful VP1/VP2 mRNA expression depends on robust viral DNA replication due to a strong internal polyadenylation site that terminates transcripts on unreplicated genomes, preventing access to the capsid gene region. NS1 expression and viral DNA replication generates the double-stranded intermediates required to bypass this site and permit VP1/VP2 transcription. Accordingly, high-level VP1/VP2 mRNA expression represents functional evidence of NS1 protein activity and robust viral DNA replication.

**Actions:** Direct quantification of viral DNA replication by bulk genome copy number measurements following transfection with the viral genome is technically challenging, because transfection introduces a large amount of input ssDNA genomes that mask replication-associated increases in genome copy number. This limitation is well recognized and precludes the interpretability of replication kinetics based solely on total viral DNA quantification after genome transfection. A comment about this was added (lines 307-308)

Following the reviewer’s recommendation, we performed immunofluorescence staining to detect the NS1 protein. The protein was detected in trophoblast cells and in the erythroid control UT7/Epo (Fig. 6D). This data provides protein-level confirmation that the B19V P6 promoter is active in trophoblast cells, thereby strengthening the conclusions of Figure 6.

We have revised the Results section corresponding to Figure 6 to remove conclusions regarding viral permissiveness and explicitly state that the transfection experiments confirm that B19V P6 promoter is active; the interpretation has been strictly limited to this mechanistic conclusion in the revised manuscript (lines 322-329)

Finally, to conclusively demonstrate productive infection, the authors should measure intracellular and extracellular infectious viral titers, for example by testing infectivity in UT7/EpoS1 cells. This point is particularly critical, as the claim that these cells are permissive for B19V infection represents arguably the main finding of the manuscript. If confirmed, it would have important implications by suggesting that B19V may productively infect cell types outside the erythroid progenitor lineage. Robust molecular and functional evidence is therefore required to substantiate this conclusion.

major points:
Figures 5 and 6
The authors should extend their analysis to include later time points post-infection or post-transfection (e.g., 120 h p.i./p.t.) to better assess the kinetics of viral replication and gene expression. Specifically, they should:
• Evaluate the production of viral antigens (NS1, VP1/VP2) by Western blotting and/or immunofluorescence (IF) to confirm protein-level expression.
• Quantify viral genome copy number over time to determine whether viral DNA replication occurs and increases at later stages.
• Assess productive infection by titrating infectious viral particles in UT7/EpoS1 cells, using both cell lysates and culture supernatants collected at multiple time points post-infection.

To date, no in vitro system, including primary erythroid progenitor cells and established erythroid cell lines such as UT7/EpoS1 or KU812, supports a fully productive B19V replication cycle with release of infectious progeny. In these canonical target cells, infection is largely restricted by intracellular blocks, resulting predominantly in non-infectious capsid production. Fully permissive in vitro conditions for B19V have therefore not been achieved.

Our trophoblast models behave analogously. While some support viral entry and early transcription, none fulfill all determinants required for productive replication. Consequently, measurement of infectious progeny is not feasible in these systems.

We further emphasize that the biologically relevant permissive environment is most likely found in first-trimester villous trophoblasts, a population for which no stable in vitro model currently exists. We therefore highlight placental organoids (recapitulate early stages of the placenta) as the most appropriate next experimental platform to address B19V replication in a physiologically relevant context.

**Actions:** As mentioned above, we have revised the manuscript to further emphasize that trophoblasts models are not permissive and that our conclusions are limited to susceptibility and early replication steps, not productive infection. Also, as mentioned above, we have performed immunofluorescence to detect the expression of NS1 protein. The results are now shown on Fig. 6D.

Figure 5
The authors should also further characterize the viral entry pathway. It would be important to clarify whether the same entry mechanism operates in placental cells and in UT7/EpoS1 cells. Sensitivity to endosomal acidification inhibitors—such as Bafilomycin A1, ammonium chloride, or chloroquine—should be tested to determine whether B19V entry is pH-dependent in both cell types.

These additional experiments would substantially strengthen the mechanistic conclusions and clarify whether the observed infection is truly productive and comparable across different cell systems.

We appreciate the reviewer’s interest in extending the work to a comparative analysis of B19V entry pathways. However, the aim of this study was to define the susceptibility of human trophoblasts to B19V by identifying the cellular determinants required for viral binding, uptake, and the initiation of early replication events. Our major findings demonstrate, for the first time, that specific trophoblast subtypes functionally express the receptor(s) required for VP1u binding and uptake (VP1uR), that globoside and proliferative activity critically shape susceptibility, and that the combined presence of these determinants converges on early villous trophoblasts as the most plausible susceptible component of the placental barrier.

Detailed dissection of the endocytic route, including pH-dependence assays, represents an interesting line of future investigation that would require a permissive trophoblast model and constitutes a separate mechanistic question not directly related to the objectives or central conclusions of the present work. We therefore consider this analysis to be beyond the scope of this study, while fully agreeing that it is an interesting topic for future research.

Reviewer #2: 1. RT-PCR of B19V NS1 and VP1/2 mRNAs: Please provide evidence that the RT-PCR products are truly from viral mRNA, and not from carry-over DNA. The DNase treatment for 1 hour does not always destroy all DNA, so a no-RT control should always be included to notice DNA carry over. Should further be mentioned whether or not these target regions were chosen to span splice sites, which is preferable, and if so, the amplicons should be sequenced (or by gel electrophoresis) to show the shorter length of mRNA-derived cDNA to that of genomic DNA. Please add at least some such evidence information to the methods section and discuss it in Discussion. Also, give manufacturer and concentration of the DNase enzyme used, there are big differences in efficiency. It is important to prove that the RT-PCR results are not from DNA, a common mistake in publications.

All RNA samples were treated with RNase-free DNase I prior to reverse transcription, and no-reverse transcriptase controls [RT(-)] were systematically included to monitor DNA carry-over. A low amplification signal was consistently detected in RT(-) controls, indicating trace residual viral DNA; this signal defined the background level used for baseline correction during analysis. All reported RT-qPCR signals exceeded the corresponding RT(-) values by a substantial margin, supporting their interpretation as deriving from viral mRNA synthesis rather than DNA carry-over. The NS1 and VP1/VP2 primer sets do not span splice junctions; therefore, the combination of DNase treatment and RT(-) controls constitutes the critical control strategy in this study.

**Actions:** RT(-) controls are now shown in the revised figures, and DNase treatment details (manufacturer, concentration, and conditions) are now specified in the Methods section (lines 664-665)

Especially in Fig 5, it seems that all cells (including REH) express viral mRNAs, up to 10E3 copies, even at 1 h pi, at which point it is supposed to have only the input virus. Line 267: “VP1/2 transcripts were undetectable” is thereby not correct. Moreover, all non-permissive cells (except REH), especially BeWo and HTR-8/SVneo cells, showed statistically more NS1 mRNA expression at 24 and 48 h than at 1 h, with ∗∗∗p < 0.001, which is even more significant increase than for the permissive UT7/Epo cells (at ∗∗p < 0.01). Where do these mRNAs come from, if the virus can neither internalize (HTR-8/SVneo and REH cells) nor reach the nucleus (BeWo, JEG-3)? Do the mRNA loads go hand in hand with the genome loads? Also, how come the signal is increasing with time in the non-permissive cells? If input has mRNA, it suggests the RT-PCR indeed shows carry-over DNA.

We thank the reviewer for this careful observation. The apparent NS1 (and VP1/VP2) signal detected at 1 h post-infection across multiple cell types is attributable to background amplification from input viral DNA, not bona fide viral transcripts. This is supported by the matched RT(-) controls, which show signals at the same background level as the 1 h samples. We have clarified this explicitly in the revised manuscript and corrected the corresponding text statements.

Regarding the statistically significant increases in NS1 signal at later time points in BeWo and HTR-8/SVneo cells, these changes occur at very low absolute copy numbers close to background and do not indicate productive infection or replication. In these cell types, the infection is restricted at entry and/or trafficking steps (for example, lack of globoside or absence of VP1uR), and we do not detect evidence for progression to replication-associated transcription.

**Actions:** We have revised the manuscript accordingly to clarify that the low viral mRNA levels observed in BeWo, JEG-3, and HTR-8 cells likely reflect minimal, inefficient viral entry via alternative mechanisms, resulting in transcription near background levels (lines 264-266).

Reviewer #3: The transfection of B19V genomes effectively eliminates the role of entry pathway-induced variation of infection. However, additional intrinsic intracellular factors affecting the progression of infection should be discussed in more detail in the discussion. Knowledge of potential intracellular bottlenecks is available in the literature.

We agree that intracellular restrictions play a critical role in determining whether B19V infection progresses beyond early steps, and we have expanded the Discussion to emphasize these points. Importantly, the progression or failure of B19V infection is already analyzed for each trophoblast subtype in the manuscript, and the underlying mechanisms are well understood:

- BeWo and JEG-3: although VP1uR-positive, they lack globoside. As shown in our study and previous work, globoside is essential for endosomal escape; thus, internalized virions remain trapped in endosomes and cannot reach the nucleus, resulting in an abortive infection.
- HTR-8/SVneo: these cells lack VP1uR, preventing viral internalization altogether. Consequently, no authentic early transcription is expected.
- hPTC^CTB^: these primary trophoblasts express both VP1uR and globoside, permitting entry, but their very low proliferative activity imposes a strong intracellular block at the level of viral DNA replication. As a result, NS1 expression is minimal and VP1/VP2 mRNAs are barely detectable.

More broadly, we emphasize in the revised Discussion that no existing in vitro model, trophoblastic or erythroid, recapitulates the full microenvironment required for a productive B19V infection. Even in primary erythroid progenitor cells and erythroid lines such as UT7/EpoS1, infection is always semi-permissive, with extensive intracellular bottlenecks and negligible production of infectious progeny. Thus, the absence of full replication in trophoblast models is not unexpected, and it is consistent with the entire B19V cell biology literature.

Our study therefore does not claim permissiveness. Instead, it identifies the determinants of susceptibility, demonstrating that placental trophoblasts possess the functional receptors and cellular context necessary for early infection steps. These insights clarify how the placenta can be a biologically relevant target for B19V, despite the lack of fully permissive in vitro systems.

**Actions:** As mentioned above, we have revised the manuscript to clarify this issue.

Authors refer to VP1uR expression in the cell lines based on an indirect method of VP1uR recognition through the internalization of its counterpart VP1u into the cells without analyzing actual VP1uR expression levels in the cells.

Please rephrase the title and text accordingly throughout the manuscript.

We fully acknowledge that the molecular identity of VP1uR is not yet known and therefore cannot be measured directly. However, the method we use, functional binding and internalization of recombinant VP1u (recVP1u) and VP1u-decorated particles (MS2-VP1u), is the established gold standard for identifying VP1uR expression. Several points support the scientific validity of this approach:

- Ligand-based detection is more specific than antibody-based detection for an unknown receptor.
  Antibodies frequently display nonspecific binding or Fc-receptor-mediated uptake, and even the availability of an antibody does not guarantee accurate receptor quantification. In contrast, recVP1u binds exclusively through the receptor-binding domain (RBD) of VP1u. Mutating this domain (ΔN29) completely abolishes binding and uptake. This makes the ligand a far more reliable indicator of receptor presence and functionality than any hypothetical antibody could be.
- recVP1u binding and internalization correlate perfectly with native B19V uptake.
  Multiple studies from our group and others have shown that VP1u-dependent binding and internalization occur only in cells that support B19V entry. The strict specificity of this assay has been validated in numerous independent cell types.
- Functional uptake demonstrates functional receptor.
  The recVP1u ligand not only binds but is internalized, demonstrating that the receptor is present and actively mediating endocytosis. This provides more information than simply detecting receptor protein levels, which would not confirm functionality.
- This concern was also addressed in response to Reviewer #1.
  We have now revised the title and text throughout the manuscript to explicitly state that we detect functional VP1uR activity, rather than claiming direct measurement of the receptor.

In summary, although VP1uR remains unidentified at the molecular level, functional ligand-based detection is the most rigorous and biologically meaningful method currently available, and it is fully consistent with established B19V entry biology.

**Action:** We have revised the title of the manuscript accordingly and changed the word “infection” for “susceptibility” (line 4).

**Part III – Minor Issues: Editorial and Data Presentation Modifications**
Please use this section for editorial suggestions as well as relatively minor modifications of existing data that would enhance clarity.

Reviewer #1: minor points

1. line 145 " These FLAG-tagged VP1u constructs were incubated with ..." I don't think incubation involved constructs (usually refers to plasmid DNA):

“FLAG-tagged VP1u constructs” refers to purified recombinant VP1u proteins carrying a FLAG epitope, not plasmid DNA. These recombinant proteins were added to cells to assess VP1u binding and internalization.

**Action:** Line 147. We have revised the text to explicitly state “FLAG-tagged recombinant VP1u proteins” to avoid any confusion.

2. line 154 " These findings reveal, for the first time, the expression of the B19V-specific receptor VP1uR beyond the erythroid lineage." I am not sure these findings actually reveal what the authors are trying to imply. Which receptor is actually used in unclear at the moment.
3. Line 170 " These results confirm the presence of functional VP1uR in CTBs from various placental origins and its absence in EVTs." Again, I am not convinced of the authors conclusion, The presence of VP1uR is not formally demonstrated. The identity of VP1uR is currently unknown, and therefore it is impossible to demonstrate its presence!

We agree that the terminology “VP1uR” requires clarification. VP1uR is defined as the cellular molecular entity targeted by the VP1 unique region (VP1u) of B19V that mediates viral binding and uptake, although its molecular identity remains unknown.

**Action:** Lines 79-81. We have now added this definition at the first mention of VP1uR in the manuscript to avoid any implication of formal receptor identification.

4. Line 253 " To determine whether B19V internalization in trophoblast cells results in productive infection, the expression of the early viral transcript NS1 was analyzed 24 h and 48 h post-infection." Detection of viral transcripts does not mean there is productive infection. To demonstrate a productive infection viral DNA must be replicated and viral particles produced.

As mentioned above, we acknowledge that detection of viral transcripts does not demonstrate productive infection.

**Action:** This point has already been addressed in detail in the sections above, and the manuscript has been revised accordingly to clarify that NS1 expression reflects early viral transcription following internalization and does not imply productive infection.

5. Line 301 " Notably, the substantial increases in NS1 and VP1/VP2 mRNA expression observed in JEG-3 and BeWo cells far exceeded the differences in transfection efficiency, underscoring the higher permissiveness of these CTB-derived cell lines to B19V replication." Again, I see no proof of B19V replication.

We agree and have revised the wording accordingly. As stated above, VP1/VP2 mRNA expression is DNA replication-dependent, whereas direct measurement of viral DNA replication after transfection is not technically feasible due to the presence of input DNA.

**Action:** As mentioned above, this sentence has been corrected. Instead of “permisiveness” we indicate “activity of the P6 promoter” (Line 292).

6. Line 307 " Overall, the findings indicate that the intracellular environment of first-trimester CTBs is significantly more permissive to B19V replication than that of EVTs or CTBs derived from term placentas." Permissiveness is not assessed.

**Action:** Lines 322-324. As mentioned above, the sentence has been reformulated to avoid the word “permissive”

Reviewer #2: 1. Please write out the abbreviations first time mentioned, very tiring to have to search the last section (Materials and Methods) for explanations, and not always finding it even there.

2. Lines 39 and 81: VP1uR, the erythroid-specific receptor” and “The VP1uR has been exclusively found in the target EPCs in the bone marrow, explaining the marked erythroid cell tropism of the virus”. How has this unknown molecule been found on EPCs and is it really specific or exclusive? In the references given, binding was searched for in only a few cell types, but not all human cells, so how can it be stated that it truly would be specific for erythroid cells? How about fetal myocardial cells, endothelial cells or liver cells? This current manuscript is actually one piece of evidence against it being specific, and there can be other cell types. Despite the heroic efforts to exclude globoside and AXL as being true cell receptors in the studied malignant cells (and earlier EPCs), there may still be a role in some cells of other known or unknown receptors, including the antibody-enhanced entry. Exact and correct wording is important so the reader would know what really are proven facts and what are not quite yet, even though perhaps likely. Perhaps using words like “has hitherto been” or “so far” or “it seems to” or similar, depending on the context and facts. The “highly restricted” on line 133 is also better in this regard.

VP1uR is defined here as the cellular molecular entity targeted by VP1u that mediates viral binding and uptake; its molecular identity remains unknown and cannot be detected with receptor-specific antibodies. Accordingly, VP1uR has been assessed operationally by VP1u ligand binding and internalization, an approach justified by the localization of the receptor-binding domain within the VP1 unique region. VP1u binding and uptake correlate with B19V binding and uptake in all cell types examined so far.

We acknowledge that terms such as “erythroid-specific” or “exclusively found” can be over-interpreted, as not all human cell types have been tested. Previous work from our group examined VP1u binding/internalization and B19V uptake in a broader range of cells, including among many others, endothelial and liver-derived cells, supporting a highly restricted expression of the cell surface molecule(s) interacting and driving uptake of VP1u. The present study extends these observations to trophoblasts.

Finally, while our data address VP1u-dependent binding and uptake as the determinant of entry, we do not exclude that additional factors may contribute in specific cellular contexts; the manuscript wording has been revised accordingly to reflect these limitations.

**Actions:** We have revised the manuscript to remove absolute statements such as “erythroid-specific”. As mentioned above, we also added the definition of VP1uR at its first mention and clarified that VP1u binding and uptake correlate with B19V binding and uptake in all cells tested so far, both in this study and in previous work from our group.

3. Line 96: Surely there are newer reviews than ref 12 from 1997, or original studies?

As suggested, we have added a more recent review (2024).

4. Line 99: perhaps use the word “risk” instead of “chance” of adverse effect.

As suggested, we have replaced “chance” with “risk” to more accurately reflect the likelihood of an adverse effect.

5. Replication per se is actually not shown, as opposed to only VP1u or virion entry, or VP protein by IF after binding or mRNA expression after transfection of B19V genomes in the various cell cultures. Viral genomes were quantified by qPCR only for Fig 4, and this was after 1 hour incubation, which is not yet replication (Lines 551-552). Mere mRNA expression is not proof of replication. If no replication experiments can be done e.g., in normal placental cells, (which would increase the impact of this paper), at least modify the wordings. Both words “infection” and “replication” are mentioned in many places, of which some do not seem to be correct. Moreover, “permissiveness” refers to a cell's ability to support a virus' complete replication cycle, not only mRNA expression after transfection. Please check that these words really mean what they should in the different contexts (including section and figure headings); is it transfection or infection and expression/transcription or replication…

This concern reflects wording rather than experimental interpretation and has been addressed above. The manuscript does not claim demonstration of productive replication or permissiveness in the trophoblast models.

**Actions:** We have revised the text to clearly distinguish binding/uptake, transcription and productive replication, and to remove terms such as “infection,” “replication,” and “permissiveness” where inappropriate. The study’s focus is now explicitly indicated as identifying where and why the B19V life cycle is blocked in trophobasts models, rather than on claiming completion of the replication cycle.

6. Lines 146-150: The cells used are all malignant immortal cell lines that may have extended viral tropisms and modified transcriptomes, which the lack of globoside proves (as said on lines 232-234 and 323-324 and 437-440 and lines 446-448). How do the authors know what is similar to the real normal placental cells? Further, Abou-Kheir et al. (Placenta 50; 2017) point out that the HTR-8/SVneo cell line contains a mixed population of cells. Further, the hPTCCTB cells are, contrary to the other cells, derived from the placental basal plate. A more detailed description of these cell lines should be included either here or in Materials and Methods, and discussed in Discussion.
The authors did obtain also normal placenta, and even cultured those cells; please mention why these were not infected with B19V (or VP1u constructs) and included in these B19V experiments (only globoside was now studied in these cells that is already known to be expressed)?

We agree that the placental cell lines used are immortalized or malignant and therefore do not fully represent normal placental cells.

Normal placental material available to us consisted of cryosections, which do not preserve cellular functionality required for viral uptake or infection assays. These samples were therefore used exclusively for globoside expression analysis, which is preserved in fixed tissue and relevant to intracellular progression of B19V infection.

**Actions:** We have added a more detailed description of all cell lines in Materials and Methods. We now also note the reported heterogeneity of HTR-8/SVneo cells (Abou-Kheir et al., Placenta 50, 2017) and clarify that hPTC^CTB^ cells are derived from the placental basal plate.

7. Line 155 says “B19V-specific receptor VP1uR”. How do the authors know this receptor is “B19V specific”? VP1uR may very well bind also other pathogens, as do globoside. Please clarify and re-write.

We agree and have changed the wording accordingly. “*These findings reveal, for the first time, the presence of a VP1u-binding receptor beyond the erythroid lineage*.” (Lines 157-158).

8. Lines 190, 700-710, 754 and 759: In the RT-PCR for globoside Gb3 and Gb4 mRNAs, what were the mRNA levels? It is only told (actually twice in the Fig 3 figure legend) that “mRNA levels were measured by RT-qPCR and normalized to GAPDH expression”. However, it is not revealed how GAPDH relates to the cell amount, i.e., how to interpret the Y axis of Fig 3, does it show the Gb mRNA copies per cell or per 1000 cells or a million cells, or what? Same for the viral mRNAs.
Line 708: Is this GAPDH quantification of mRNA or DNA? qPCR would mean DNA. Did you have a positive control for mRNA? Please use italics for gene names to differentiate mRNA from the actual protein product (for NS1, VP1/2, Gb, GAPDH and Epo)

Gb3, Gb4, and viral transcripts were quantified by RT-qPCR at the mRNA level and normalized to GAPDH mRNA, which serves as an internal reference for RNA input and cell number. The Y-axis values therefore represent relative mRNA abundance normalized to GAPDH, not absolute copy numbers per cell. GAPDH was quantified exclusively as mRNA after reverse transcription (not genomic DNA), with appropriate RT(–) and positive mRNA controls as described above.

**Actions:** We have clarified in the Methods section and figure legends that all RT-qPCR data are presented as relative mRNA levels normalized to GAPDH mRNA, and that GAPDH was measured at the RNA level. The manuscript text has been reviewed to ensure consistent and unambiguous distinction between transcript measurements and protein detection.

9. Lines 239-240 and Fig 4: Does trypsin really remove the B19V capsids? Are there proof of this? Does trypsin cleave either the capsid or the VP1uR? In Fig 4 the cells look the same with and without trypsinization. Please explain.

Trypsinization is a standard method to remove surface-bound, non-internalized viral particles. Its effectiveness was validated in UT7/Epo cells incubated with B19V at 4 °C, a condition that allows viral binding but prevents internalization; under these conditions, trypsin efficiently removes cell-associated capsids. At 37 °C, viral binding and internalization are rapid and tightly coupled processes. After washing to remove unbound virus, the majority of virions have already been internalized, while only a small fraction is transiently present at the cell surface at the moment of stopping the incubation. Consequently, most cell-associated viral genomes are resistant to trypsin treatment, resulting in only minor differences between trypsinized and non-trypsinized samples in Fig. 4, which are further compressed by the logarithmic scale.

**Actions:** We have now added the trypsin control to Figure 4.

Top of Form

Bottom of Form

10. Line 245-249 and Fig 4: “A strong viral signal was observed in BeWo cells, weaker staining in JEG-3 and hPTCCTB, and no detectable signal in HTR-8/SVneo”. However, this is not correct; in Fig 4B there is no signal in hPTCCTB cells either, please correct.

We thank the reviewer for noting this discrepancy. Although a very weak viral signal was detectable in hPTC^CTB^ cells by immunofluorescence, it is not visible in the representative images shown in Fig. 4B.

**Actions:** To avoid confusion and ensure consistency between the text and the figure, we have revised the manuscript to describe the signal in hPTC^CTB^ cells as undetectable (Line 252).

Line 248: Fig 1 does not really show “levels of VP1uR expression” (there is no RT-PCR for VP1uR), but instead it shows binding of VP1u, which should correlate with the presence of its receptor. This wording may confuse the reader, so best to be specific and exact with the wording.

**Actions:** We have corrected the wording to replace references to “VP1uR expression” with VP1u binding and uptake, clarifying that Fig. 1 and 2 report functional VP1u ligand binding as a proxy for the presence of a VP1u-binding receptor, rather than direct measurement of receptor expression (253-255).

11. Line 272: How many % of cells were “infected” in each cell culture, did the yield correspond to the % infected? This would need to be calculated, instead of normalising to GAPDH expression that occurs in all cells.

RT-qPCR normalized to GAPDH is a standard approach to quantify relative mRNA expression at the population level. It does not measure, and is not used here to infer, the percentage of infected cells; rather, it allows comparison of transcriptional output across cultures under identical conditions.

12. Line 275 and 346: What is “structural mRNA expression”? Please correct the wordings.

By “structural mRNA expression,” we refer specifically to the expression of the structural (VP1/VP2) mRNAs.

**Actions:** We have revised the wording accordingly to make this explicit and avoid ambiguity (Lines 280 and 360).

13. Lines 284-315 and Fig 6: Transfection efficiencies were quite low and differed between cell types. The expression of e.g., hPTCctb could proportionately be much higher if taking into account that only a small fraction of cells was transfected. Same stands for the infection efficiencies in the various cells (see comment 11). Were the expression levels calculated for the proportion of transfected (or infected) cells or all cells? If the latter, there is a bias depending on the fraction of cells that contain the virus genome, so this should be calculated, or at least discussed.
On line 302-304 this subject is approached, but it is not really understood, and the math is lacking: The B19V non-permissive JEG-3 and BeWo cells have both higher transfection efficiencies and higher viral mRNA expression efficiencies than the B19V-permissive UT7/Epo cells, which proves my point, the actual transfected cells may have high transcription rate but the overall transcription is still low because too few cells have been transfected.
Furthermore, the JEG-3 and BeWo cells cannot be described as B19V permissive, as now is indicated on line 303?? However, another statement later (lines 314-315) summarizes that these cells are indeed resistant to B19V infection, but the reader may be confused if the wording is not carefully chosen…] The heading of Figure 6 is thus also misleading in two places: “Permissiveness of trophoblast subtypes to B19V infection.” (see comment 3 above) Please correct.

Viral mRNA levels in Fig. 6 were quantified from bulk cell populations and represent average transcript levels across all cells, not per transfected cell. Transfection efficiency was measured independently and considered in the interpretation, and the data are not used to infer permissiveness or per-cell transcriptional capacity.

**Actions:** We have clarified in the Results section that viral mRNA measurements in Fig. 6 represent population-level transcriptional output and are not normalized to transfection efficiency, and revised the corresponding text, section heading, and figure title to remove any implication of permissiveness or productive infection (Lines 290, 301-303, 805)

14. Line 326: Polyethyleneimine is misspelled.

The spelling of polyethyleneimine has been checked and is correct.

15. Line 360: How was the Epo mRNA quantified?

The quantification of Epo mRNA was performed by RT-qPCR following RNA extraction, DNase treatment, and reverse transcription, using Epo-specific primers and normalization to GAPDH mRNA. This was implicit in the original text but is now stated explicitly to avoid ambiguity.

**Actions:** We have clarified in the Methods section how Epo mRNA was quantified, including the RT-qPCR procedure and normalization strategy (Lines 729).

16. Lines 371-372 and 466: This interpretation is perhaps not the full truth. This may also suggest that BeWo cells do not need Epo due to the malignant transformation and rapid growth, but perhaps normal trophoblasts, that truly are dependent on Epo (like UT7/Epo cells are), would need Epo also to permit B19V replication? Difficult to use malignant BeWo cells for studying normal placenta behaviour (see comment 6 above).

Both BeWo and UT7/Epo cells are malignant and highly proliferative; however, they differ fundamentally in their dependence on Epo. UT7/Epo cells remain strictly Epo-dependent for proliferation and survival, and B19V replication in these cells is likewise Epo-dependent. In contrast, BeWo cells proliferate independently of Epo, likely as a consequence of malignant transformation. Our interpretation is therefore not that Epo signaling is irrelevant for B19V gene expression per se, but that high proliferative activity alone is insufficient to support replication in BeWo cells, despite their Epo independence. Whether normal trophoblasts, which may retain Epo responsiveness, require Epo signaling to permit B19V replication cannot be addressed with the current models and remains an open question.

**Actions:** We have added the sentence “*Nevertheless, the lack of Epo dependence observed in trophoblast models does not exclude a role for Epo signaling in the context of the placenta*” to the Discussion to explicitly limit extrapolation from trophoblast models to the placenta (485-487).

17. Line 394: This sentence should be “These complications are more frequent when infection of the mother occurs…” Those references, and others, say that the highest risk for fetal death is when the mother is infected during the first half (20 weeks) of pregnancy, after which the excess risk goes to zero. But 80% of fetal deaths occur within 4 weeks and almost all within 12 weeks of maternal infection (Enders et al. 2004). The lines 457-458 may thus need to be slightly modified, unless more proof is given of first-trimester fetal infection affecting the fetus more severely than middle-trimester fetal infection, i.e., differentiating maternal and fetal infections. Of course this may be true, because the early fetus is generally more vulnerable, but it would help the case if some proof is provided from the literature.

We revised the relevant sentences to clarify that risk is linked to the timing of maternal infection rather than fetal gestational stage. References to first-trimester infection were replaced with wording reflecting the higher risk associated with earlier maternal infection, in line with epidemiological evidence.

**Actions:** We revised the wording to reflect maternal infection timing and align the text with the cited literature (Lines 408-409, 472).

18: Line 423-432 and 471-478: It is stated that globoside is used as B19V receptor in acidic conditions (airways) and VP1uR in neutral conditions, such as bone marrow. However, no pH measurements have been done in this study for the placental cells? Normally the placenta is known to be slightly alkaline, which corroborates the use of VP1uR, yet only globoside was studied in the human placenta, but no pHs, so please clarify why and add placental pH aspect to the Discussion.

The pH dependence discussed refers to endosomal acidification, not extracellular placental pH. VP1uR mediates viral uptake at the cell surface under neutral conditions, whereas globoside engagement occurs intracellularly in acidic endosomes, a conserved process that does not require direct pH measurement. The model integrates published data and does not imply differences in placental extracellular pH.

19: Line 449: “Our findings indicate that both CTB and STB populations are susceptible to B19V.” Please elaborate which exact cells are here referred to and which experiments indicate this. Also, clarify in the text if “susceptible to B19V” here means replication after B19V infection or only transcription after B19V-DNA transfection?

“Susceptible” refers to a cell that supports viral binding and entry, not productive replication. The statement refers specifically to villous cytotrophoblast–derived cells (BeWo, JEG-3, hPTC^CTB^) and syncytiotrophoblasts generated by forskolin-induced differentiation, which express the receptor(s) required for VP1u interaction and B19V entry, in contrast to extravillous trophoblasts (HTR-8/SVneo). This conclusion is supported by VP1u binding/uptake assays and virus uptake experiments and does not imply viral replication.

**Actions:** We have revised the wording to specify the exact trophoblast populations analyzed and to clarify that “susceptible” refers to viral binding and entry, not productive replication (Line 464).

20. Line 459: The highly proliferative BeWo and JEG-3 “which originate from first-trimester trophoblasts” could also be fast growing due to malignancy, and not for being from the first trimester. AS mentioned, they are not physiologically relevant as model cells. This would be good to be discussed.

The reviewer is correct that the high proliferation of BeWo and JEG-3 cells may partly reflect their malignant origin. However, this does not contradict our interpretation. Primary first-trimester villous cytotrophoblasts are intrinsically highly proliferative compared to term CTBs, a well-established physiological feature of early placental development. B19V replication is known to require cells in active proliferation to support viral DNA replication. Accordingly, our interpretation does not rely on malignancy per se, but on the convergence of high proliferative capacity, which is shared by both malignant first-trimester CTB-derived models and normal early villous CTBs, and is absent in term CTBs, as exemplified by hPTC^CTB^ cells. Thus, the limited viral progression observed in hPTC^CTB^ cells is consistent with their reduced proliferative state rather than with differences in malignant transformation.

**Actions:** We have changed the sentence in line 459 accordingly “…the first-trimester trophoblasts BeWo and JEG-3, whose high proliferation is further supported by their malignant nature, produce significantly higher levels of NS1 mRNA upon transfection than term placenta-derived hPTC^CTB^ cells.” (Lines 474-475).

21: Line 480-481: “this study demonstrates that B19V can enter and infect placental trophoblasts”: This study actually shows on one hand, that the virus genome can be transfected into some immortal placental cells leading to transcription and translation, and on the other hand that that virions can enter some but not all cells, but to be strict, it does not really show that the B19 virus can infect, transcribe, replicate and produce progeny viruses in true placental trophoblasts. If really not able to infect those natural placental cells, it would be good to at least explain why 1+1 must be 2.

As the reviewer indicates, the study does not demonstrate a complete productive B19V infection cycle in any single model. Although no in vitro system recapitulates all requirements simultaneously, this limitation applies equally to erythroid progenitor cells and erythroid cell lines, which are likewise only partially permissive. Our data suggest that the essential determinants of B19V infection converge in early villous trophoblasts.

**Actions:** As noted above, we have revised the wording throughout the manuscript to clarify that specific trophoblast subtypes support viral entry and transcription, rather than full productive infection, and to align all conclusions accordingly.

Top of Form

Bottom of Form

22: There is a lot of methodology included in the Results section, but it still does not include everything, so please refer in Results to the Materials and Methods section if more details are given there, and vice versa, include in short under the Materials and Methods subheadings, which experiment used which method.

**Actions:** We have improved cross-referencing between the Results and Materials and Methods sections. In the Results, we now refer explicitly to the corresponding Methods for experimental details, and in the Materials and Methods we clarify which experiments and figures each method was used for.

Reviewer #3: To increase the readability of the paper the cells lines mentioned in the text should be in the same order as in the figures e.g. Fig 1, Fig 3,

We have reviewed the manuscript and ensured that, wherever possible, cell lines are presented in the same order as in the corresponding figures to improve readability.

The results of the supplementary Figure S1 A, B, and C are not adequately explained in the results.

As suggested, we have expanded the Results section to more clearly explain the data presented in Supplementary Fig. S1A-C (Lines 182-189).

In Fig 2B and Fig 3B the multinucleated syncytia formation of hPTCCTB cells due to STB differentiation with forskolin is not visible from the images. This should be shown, as syncytia formation is used as a marker for successful differentiation.

We acknowledge that multinucleated syncytia are not prominently visible in the representative images shown in Fig. 2B and Fig. 3B. However, closer inspection reveals cells containing multiple nuclei within a shared cytoplasm, consistent with syncytial differentiation. In Fig. 2B, a large cell with two nuclei displays a clear VP1u-associated signal, and in Fig. 3B, cells with multiple nuclei are surrounded by a continuous globoside-positive signal. In addition, forskolin-induced differentiation of trophoblasts into STBs is a well-established and widely validated approach and was applied here following standard protocols.

The beginning of Figure 4 legend contains text that should be in the Materials and Methods section. Please modify.

We have revised the Figure 4 legend to remove methodological details that belong in the Materials and Methods section. The legend now focuses on describing the data shown and how to interpret the figure, while experimental procedures are referred to the Methods section.

In Figure 7E. The amount of VP1/VP2 mRNA in BeWo cells at 24 and 48 hpi is 0 in the absence of PEI. This is confusing when compared to Fig. 4. Could you please explain in detail?

We note that this comparison refers to Fig. 5B rather than Fig. 4. The apparent discrepancy reflects very low background VP1/VP2 mRNA signals and differences in y-axis scaling, not a biological difference. In BeWo cells without PEI, VP1/VP2 mRNA levels remain at background in both figures (~10² in Fig. 5B and <10 in Fig. 7E). Because the y-axis in Fig. 7E starts at 10, these values are not visible and appear as zero.

Fig.8C, E. The text on top of the diagrams is too small.

As suggested, the text size in Fig. 8C and Fig. 8E has been increased to improve readability.
